# Cytodiagnosis of Basal Cell Adenoma of Parotid Gland: A Rare Case Report

**DOI:** 10.7759/cureus.36767

**Published:** 2023-03-27

**Authors:** Poornima Pandey, Arvind Bhake

**Affiliations:** 1 Pathology, Jawaharlal Nehru Medical College, Datta Meghe Institute of Higher Education and Research, Wardha, IND

**Keywords:** nodular swelling, diagnosis, salivary neoplasms, basaloid cells, cytomorphology, immunohistochemistry, fine needle aspiration cytology, parotid gland, basal cell adenoma

## Abstract

Basal cell adenoma is a rare benign neoplastic lesion of the parotid gland, therefore it is rarely diagnosed in preoperative work-up by fine needle aspiration cytology. This distinctive entity being regarded as one of the scarce salivary neoplasms is predominantly perceived in the female population, but is uncommon in young adults. It is extremely challenging to diagnose basaloid tumors predominantly basal cell adenoma of the salivary gland on cytopathology. Therefore present case report describes the fine needle aspiration cytology diagnosis of basal cell adenoma in the left parotid gland in a 40-year-old female. The case report also describes the cytomorphological characters, the cytological differential diagnoses and immunohistochemistry of basal cell adenoma.

## Introduction

Basal cell adenoma (BCA) is a rare benign salivary gland tumor. It constitutes about 1-3% of all salivary gland neoplasms [1,2]. It presents clinically as the nodular swelling in pre- or infra-auricular area in the parotid gland. However, its location and consistency are perplexing for surgeons for its wide spectrum of differential diagnoses. Its occurrence is infrequent, therefore becomes a clinical as well as diagnostic novelty [2,3].

Fine needle aspiration cytology (FNAC) of nodular swellings in superficial as well as deep lobes of parotid glands by now is a popular diagnostic modality. The reports in the literature that describes the diagnosis of BCA in the parotid gland are rare. It is therefore all more interesting for its cytomorphology and cytological differential diagnosis [2-4].

The diagnostic dilemma on the cytological preparation may be a limiting factor for the surgical treatment of these lesions [2,3]. The present case report describes the cytodiagnosis of a BCA carried out on FNAC in an adult female with certain interesting clinical features.

## Case presentation

**Figure 1:**
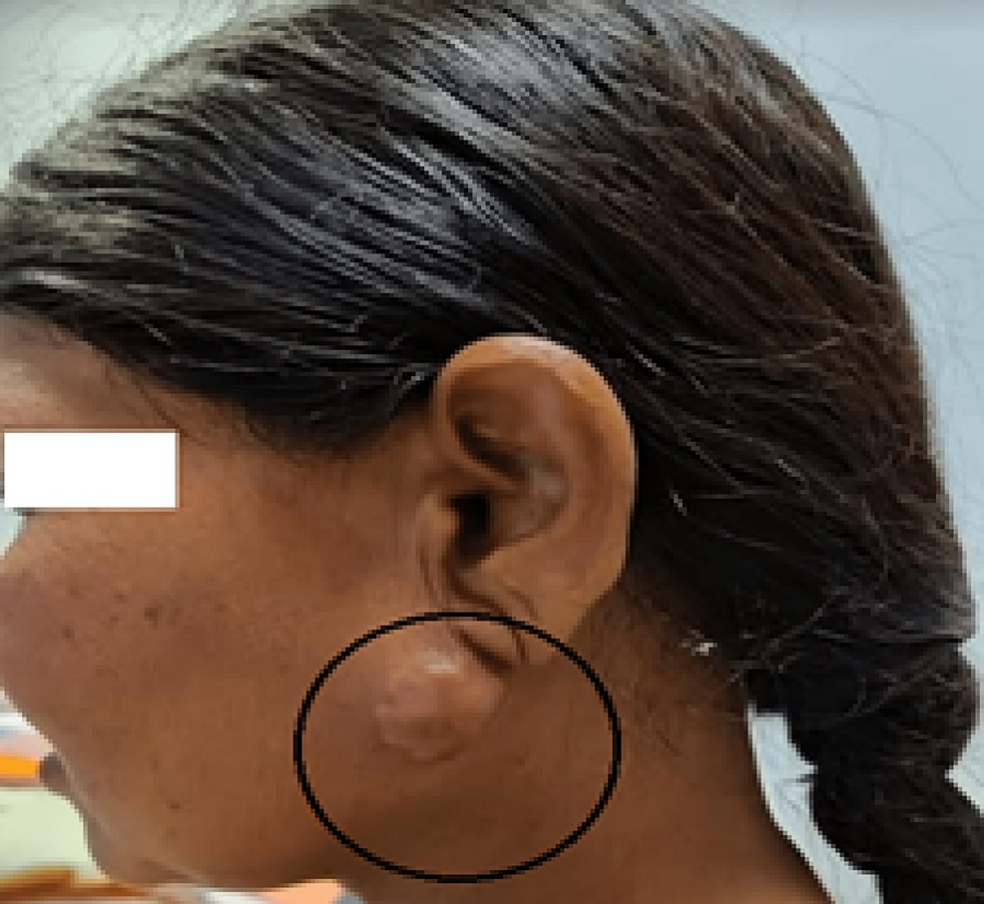
Clinical photograph showing nodular infra-auricular swelling in the parotid region with a little scarring and hypopigmentation of skin overlying the swelling.

A 40-year-old female came to the surgery Out Patient Department with complaints of disfiguring nodular swelling in infra-auricular area of face. The skin over the swelling appeared hypo-pigmented. The history revealed that the patient had applied some locally available medicinal paste over the swelling that bought this change of hypopigmentation and a little of scarring over the skin (Figure 1).

The patient complained of the swelling since two years which was of insidious onset without significant pain in it. Lately she started complaining that the swelling on palpation is little firmer.

The local examination carried out showed the following findings: i) swelling was located on left side of face in parotid region, ii) swelling was measuring 2 x 2 cm, iii) firm in consistency with restricted mobility, iv) non-tender and v) non-cystic on examination.

The patient was admitted in surgery ward for the treatment of swelling. She was referred to the division of cytopathology for FNAC, meanwhile she underwent the preoperative baseline laboratory investigations. The complete blood count was within the normal limits including her red blood cell indices. The prothrombin time/international normalized ratio (PT/INR) of the patient was 1.11. Her urine examination for microscopy and routine chemical tests were normal. Her urea and creatinine were within normal limits and so were the electrolytes. The liver enzymes alanine transaminase (ALT) and aspartate aminotransferase (AST) too were within normal range, so were albumin and bilirubin levels. Her random blood sugar level was 97 mg/dl. She underwent the estimation of T3, T4 and thyroid stimulating hormone (TSH) which was found to be normal. A pre-operative virological work-up for hepatitis B surface antigen (HbsAg), HIV and hepatitis C virus (HCV) was of non-reactive status.

**Figure 2:**
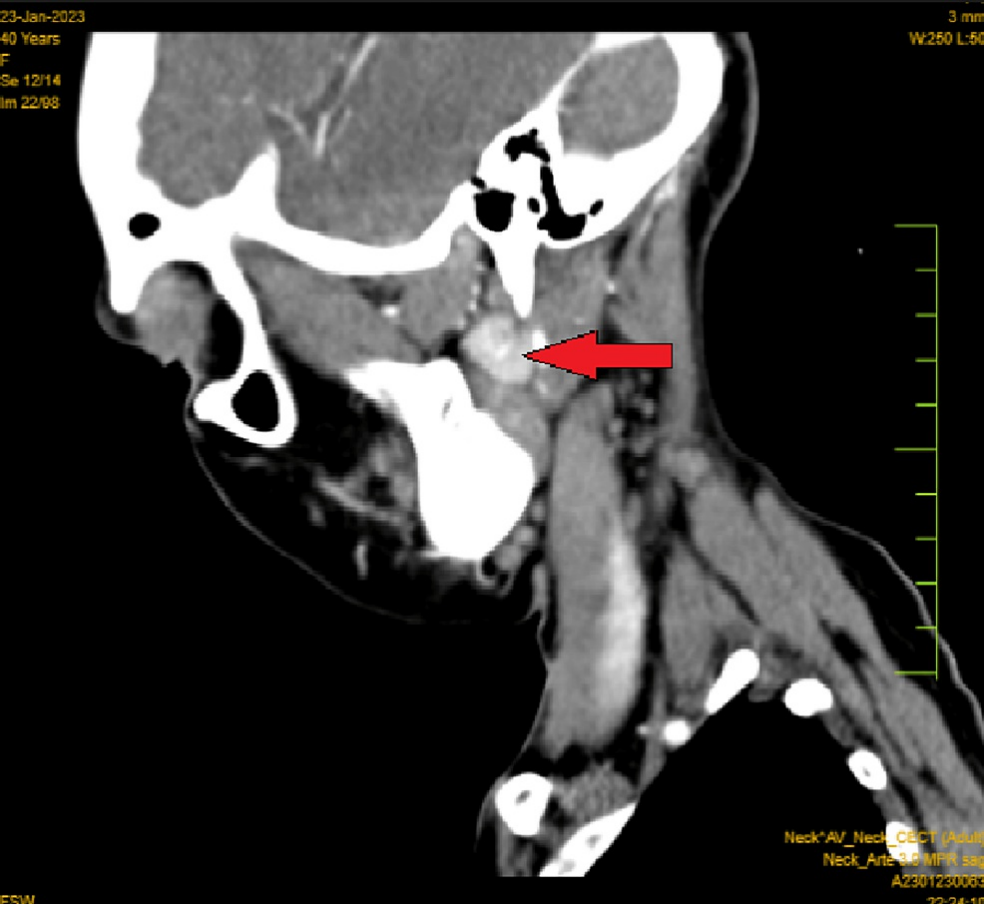
CT Head and Neck (Sagittal section)

The CT scan of head neck region was performed. The sagittal section showed a round, well defined iso-attenuated mass measuring 2 x 2 cm in the superficial lobe of left parotid gland with homogenous enhancement. The white arrow points to homogenously enhancing mass in superficial lobe of the left parotid gland (Figure 2).

The FNAC was carried out by 24 gauge hypodermic needle by standard procedural steps. The aspirate obtained was dry fixed and wet fixed in 95% ethyl alcohol. The dry fixed smears underwent May-Grunwald-Giemsa (MGG) stain while the wet fixed smears were stained by Papanicolaou stain. The standard references to report salivary cytology were used [5].

**Figure 3:**
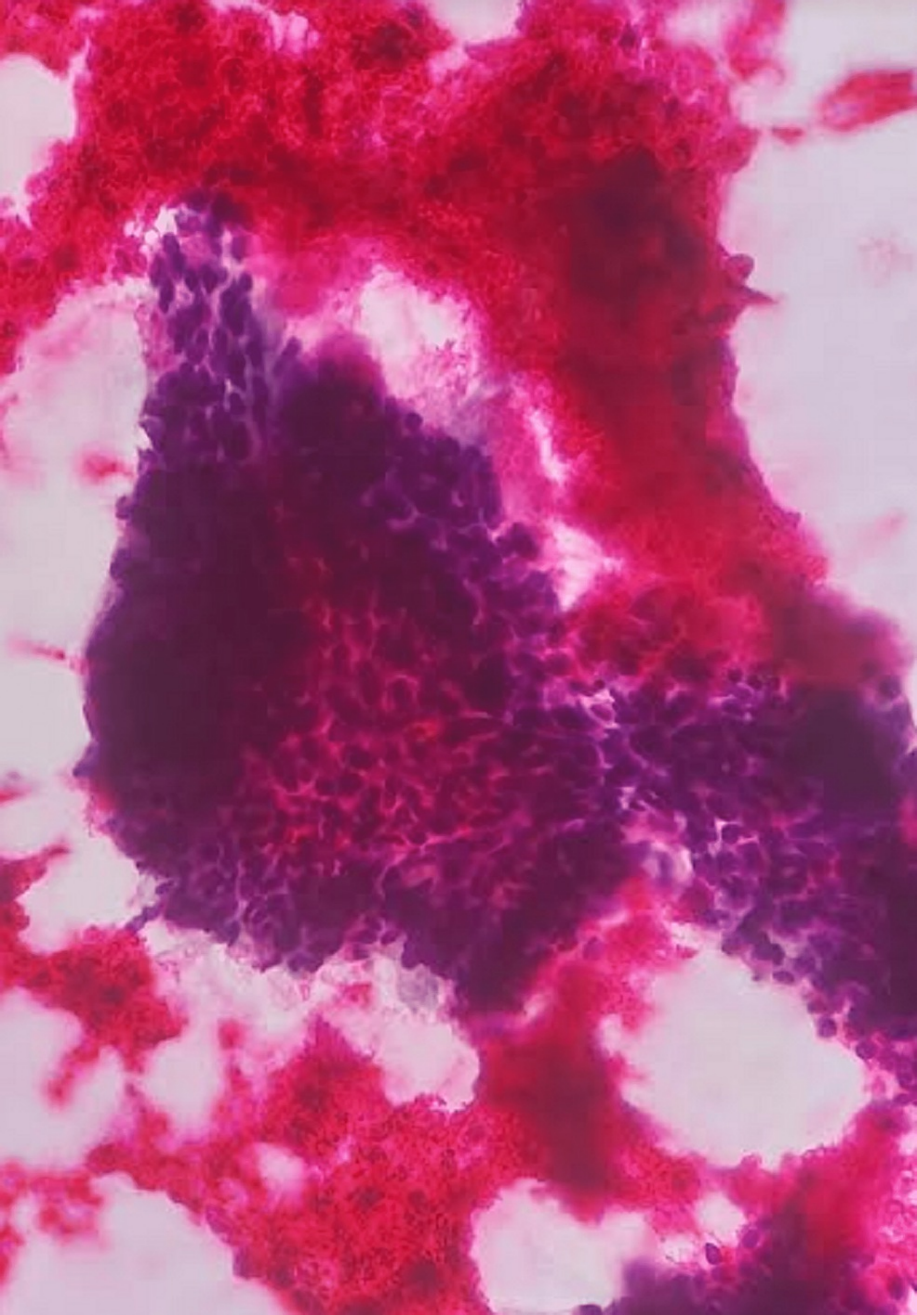
Basal cell adenoma fine needle aspiration cytology (FNAC), Papanicolaou stain - 40x

**Figure 4:**
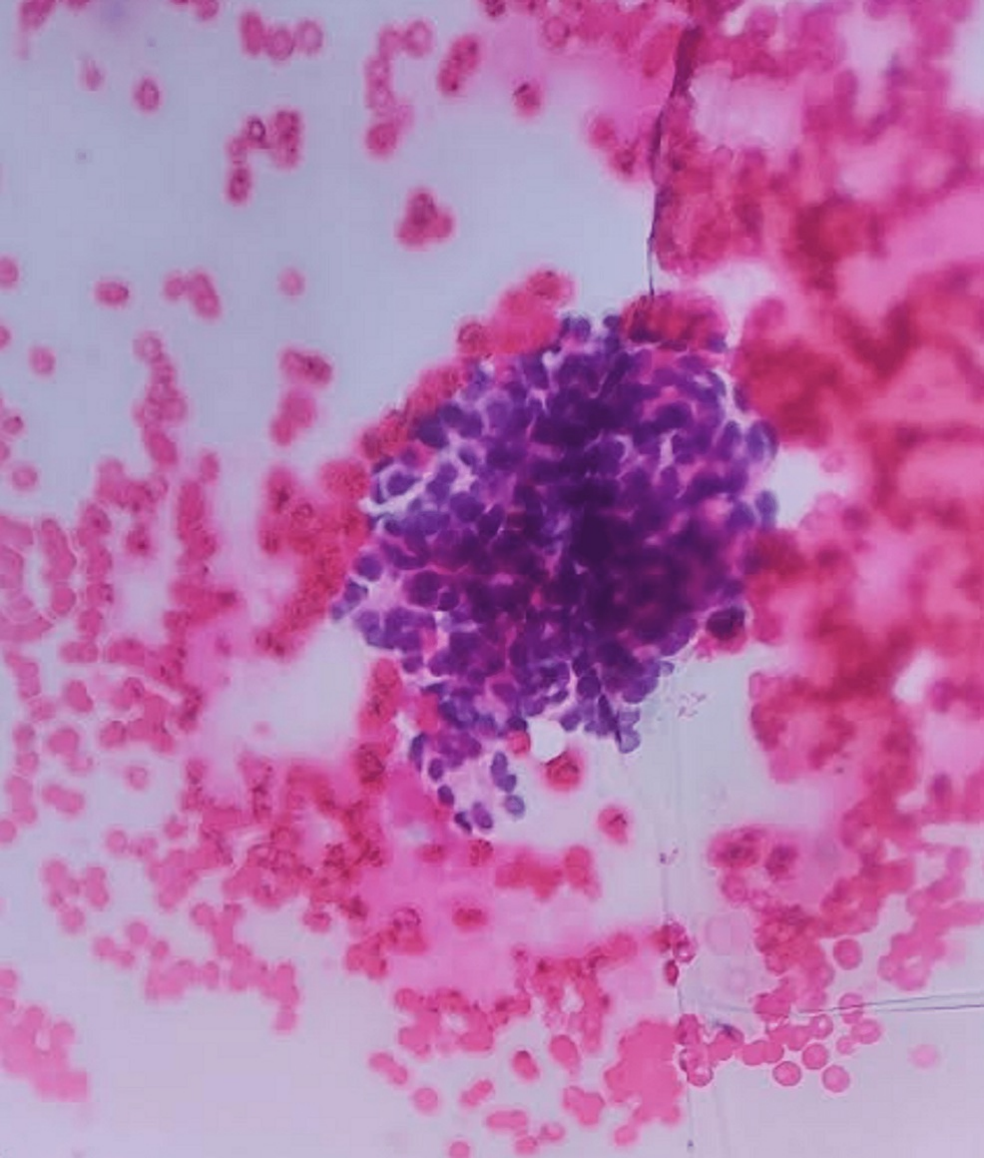
Basal cell adenoma fine needle aspiration cytology (FNAC), Papanicolaou stain - 40x

The smears were cellular. Smears showed multiple adenoid cylinders of basaloid cells which were placed cohesively. At places these cells were forming globular rounded cell placement separated by the strands of vascular stroma. The cells were cuboidal-basal like with polar nuclei. Nuclei were comparatively darker than the rest of normal ones with variability in size, shape and texture. A few of these cell sheets showed nuclear streaming. The cytoplasm was scant to modest with cuboidal morphology. A few spindle stromal cell fragments too were seen. Cytomorphology was interpreted as basal cell adenoma, in which there was absence of other mesenchymal components, basement membrane material or hyaline globules which helped to differentiate from its membranous subtype which is constituted by compactly placed cells lined by an intense hyalinized basal membrane. No cytologically malignant cells were seen. A sheet of basaloid cells placed cohesively in rounded adenoid cell group with nuclei that lacked atypia is shown, Papanicolaou stain - 40x (Figure 3) and a rounded group of cuboidal basaloid cells with peripheral palisading is shown, Papanicolaou stain - 40x (Figure 4).

**Figure 5:**
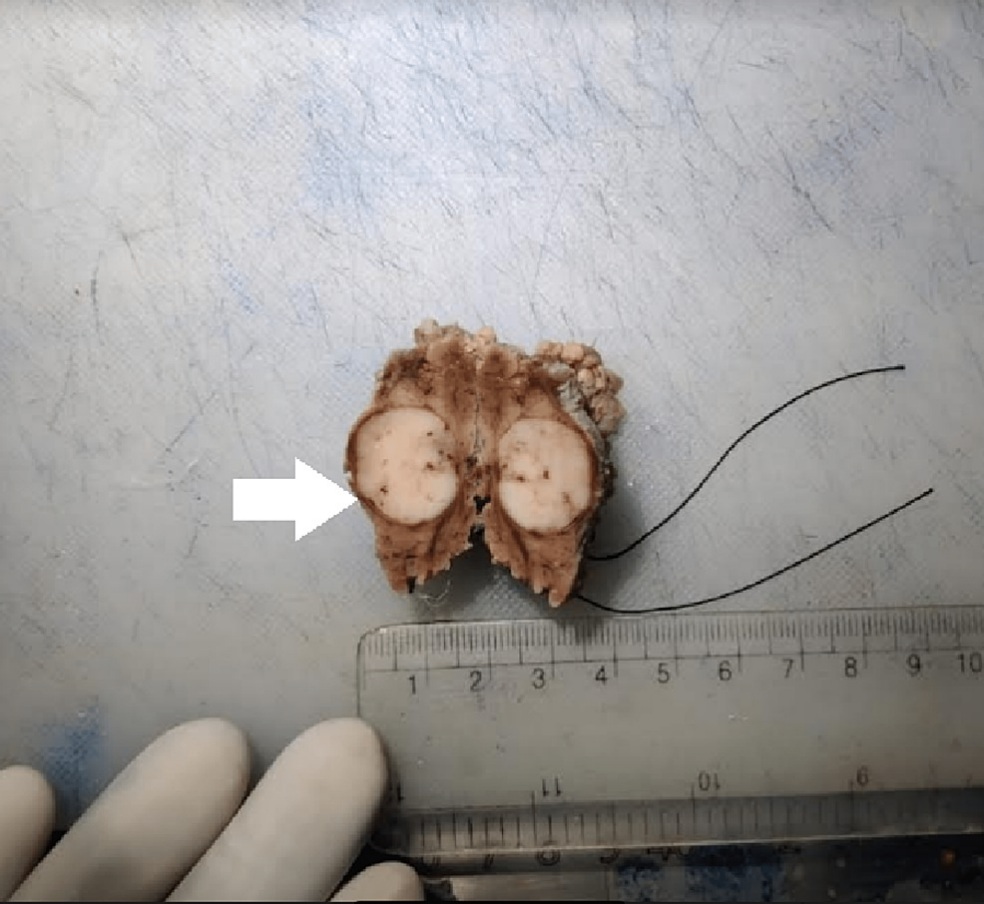
Gross (bisected specimen)

Upon the reports of the cytology, patient underwent partial parotidectomy of the mass from the superficial lobe of parotid gland. The whole of the resected specimen was measuring 3 x 1.9 x 1.9 cm sustaining a mass measuring 2 x 2 cm. The cut-section was solid, grey white. It showed a well-circumscribed lesion with no cystic degeneration except for small punctate hemorrhages. The lesion was surrounded by normal appearing grey fleshy salivary tissue with a well circumscribed nodule in center which appeared encapsulated (Figure 5).

**Figure 6:**
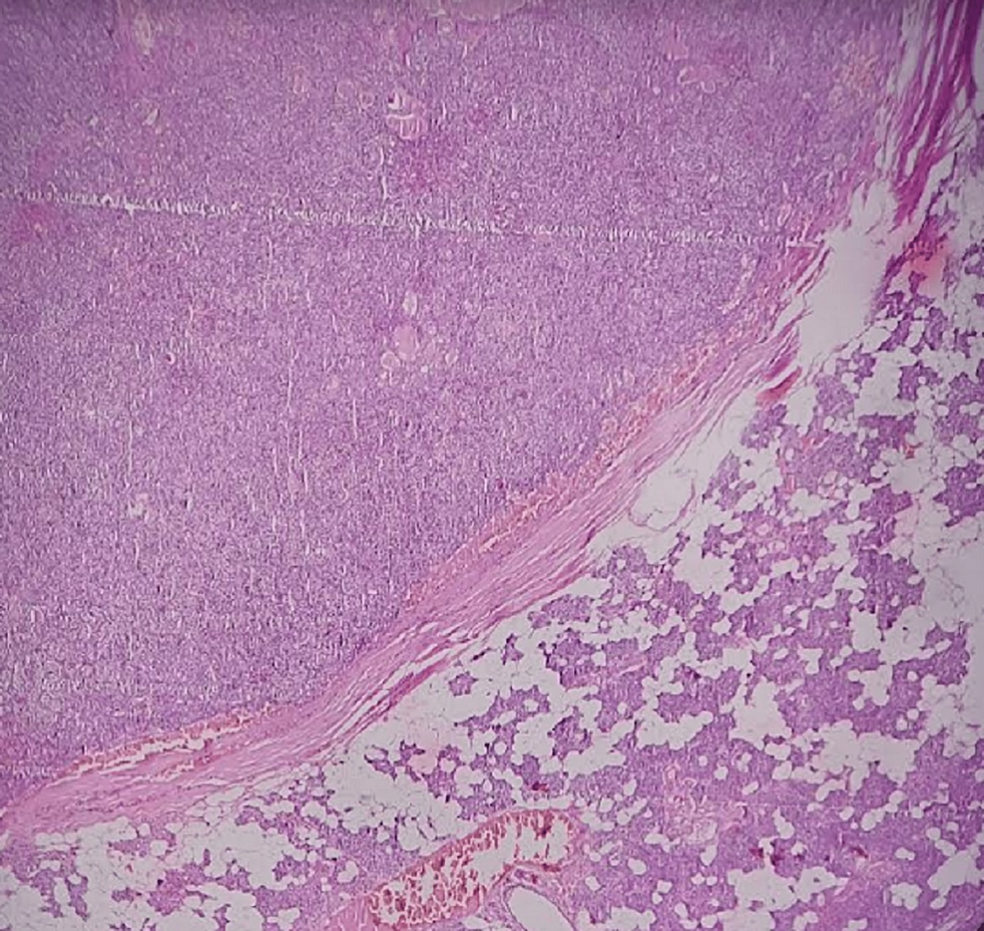
Basal cell adenoma (Histopathology), H and E - 4x (Scanner)

**Figure 7:**
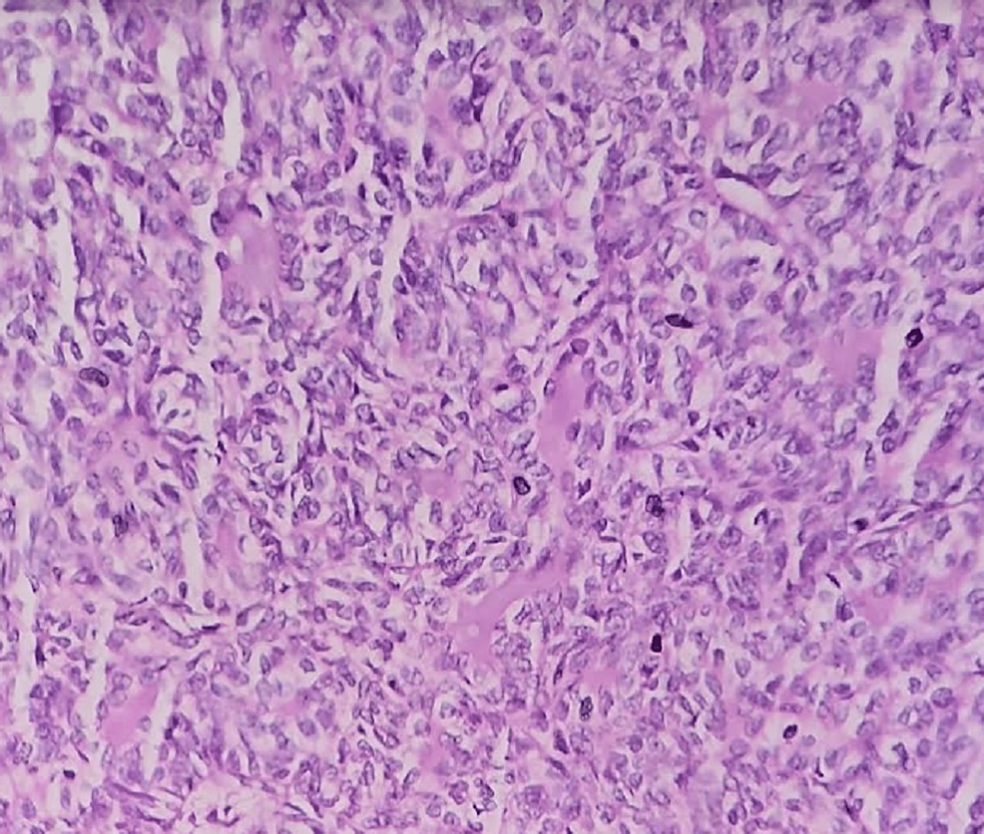
Basal cell adenoma (Histopathology), H and E - 40x

The sections from the tumor as well as tumor margins and from normal salivary gland tissue were taken. These tissue sections were later processed in automated histokinette to make paraffin sections. The tissue paraffin sections were stained by hematoxylin and eosin (H and E). Section showed a capsulated tumor surrounded by normal salivary gland tissue. The tumor consisted of benign basaloid epithelial cells with histomorphology consistent with basal cell adenoma of parotid. An encapsulated lesion distinct from surrounding normal salivary acinic cells, H and E - 4x is shown in Figure 6 and adenoid structure made of basal cells with polar nuclei separated by vascular collagen strands, H and E - 40x is shown in Figure 7.

**Figure 8:**
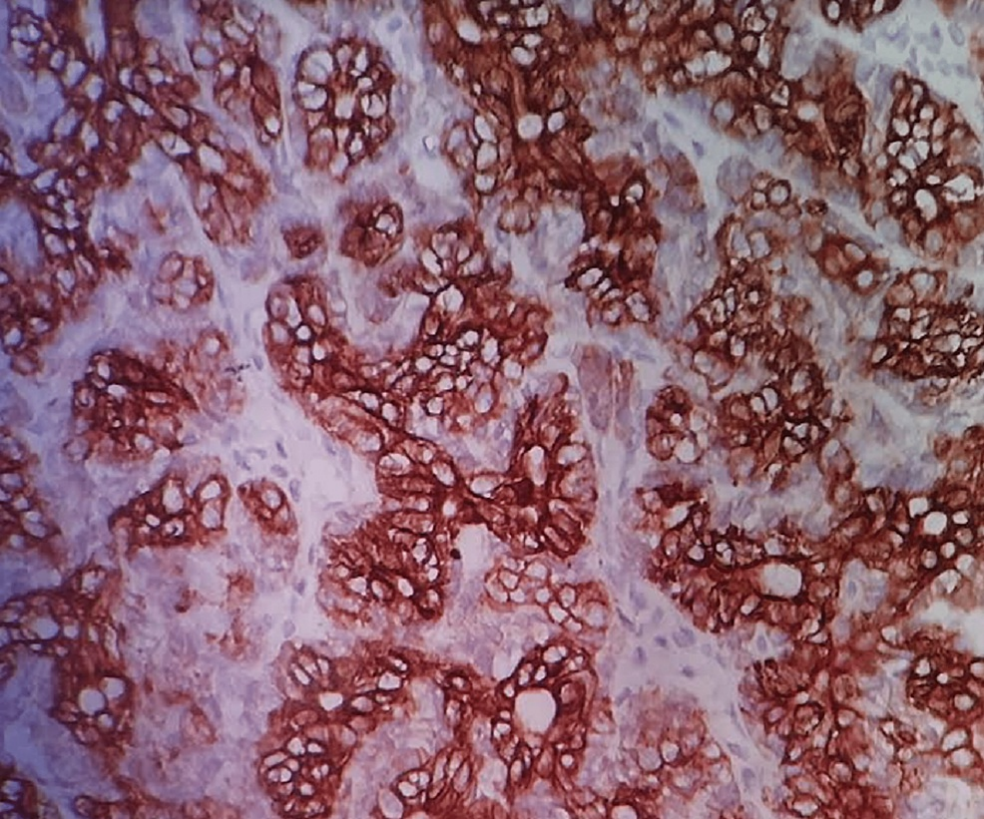
Basal cell adenoma (Immunohistochemistry, PAN-CK - 40x)

**Figure 9:**
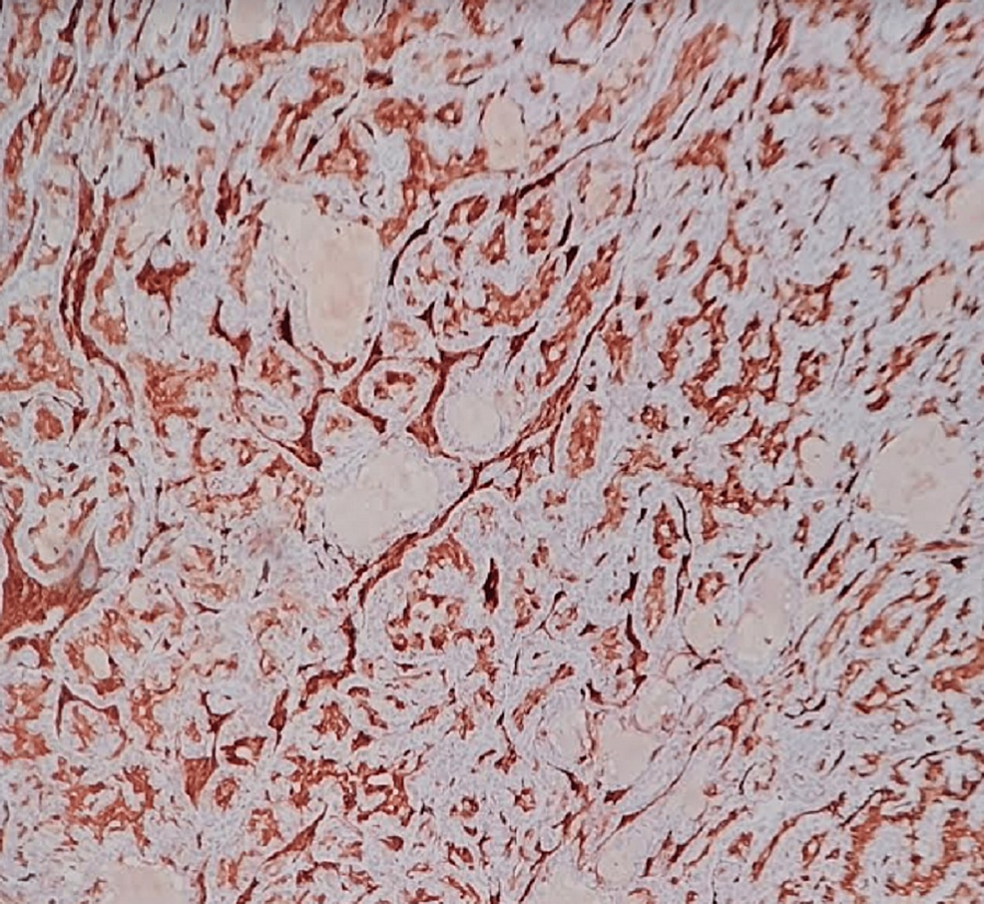
Basal cell adenoma (Immunohistochemistry, S100 - 10x)

The sections from tumor underwent immunohistochemistry for pan-cytokeratin (PAN-CK) which showed positive results. There was intense brown cytoplasmic granularity seen in tumor cells (Immunohistochemistry [IHC], PAN-CK - 40x) shown in Figure 8. The immuno-staining for S100 protein in the proliferating cells of tumor was variable and a part of proliferative tumor cells were negative and a part of basaloid cells were positive (IHC, S100 - 10x) as seen in Figure 9.

## Discussion

BCA in the parotid gland is a rare benign epithelial neoplasm. Conventionally the adenomas of the parotid gland are classified either as pleomorphic adenoma or adenoid cystic carcinomas [6]. BCA has a morphology that is monomorphic for cellular character [7-9]. Isolated reports exist in literature which describe the histomorphology and its immunohistochemistry [10].

However, the cytological diagnoses of BCA performed on the FNAC material from lesions in parotid are rarely reported in literature [1,3,4]. The cytomorphological differential diagnoses of the BCA are due to the small size of the cells, the cohesiveness of the cells and the nuclei with subtle atypia [3].

Most of the published reports mention adenoid cystic carcinoma and cellular pleomorphic adenoma as differential diagnoses of BCA on the cytologic preparations [1,3,4]. The exclusion of aforesaid differential diagnoses can be made on the cytology by presence or absence of certain cytomorphological characters. The cellular pleomorphic adenoma in their aspirate contains the other mesenchymal component such as fibromyxoid flakes, chondromyxoid flakes, presence of bare nuclei and the other components comprising a mixture of polygonal epithelial cells and myoepithelial cells laid in a matrix of mucoid or hyaline in origin co-exist in the lesion of pleomorphic adenoma. These cytological features are absent in smears of BCA. The cytology of adenoid cystic carcinoma shows excessive finger-like laying of basement membrane material in between the organoid groups of small epithelial cells, so also there is presence of hyaline globules and a cartner wheel morphology of cell placement. These features are absent in cytologic smears in diagnosis of BCA.

The present study observed the monomorphic population of basaloid cells which are small cuboidal and placed cohesively. The cells are seen to form the adenoid small ball-like structure within the sheets as well as in isolation. These cell sheets are possessing globoid shapes with peripheral palisading arrangement of the tumor cell nuclei. The large cell size clusters with three-dimensional orientation possessing scant stromal components too were seen in the present case. The nuclei were a little hyperchromatic but lacked atypia or pleomorphism. The diagnosis of BCA could be entertained by virtue of monomorphy of the cells and above features. Vicandi et al. [1], Hara et al. [3] and Kawahara et al. [4] have reported the diagnosis of BCA in parotid by FNAC. The reports of Nakabayashi et al. [6], Kanaujia et al. [7], Singh et al. [10] and Reddy et al. [11] have reported erroneous and inconclusive cytological diagnosis of BCA on FNAC which on histological examination turned out to be basal cell adenocarcinoma, adenoid cystic carcinoma, pleomorphic adenoma and basaloid squamous cell carcinoma respectively. These authors reported it due to the overlapping cytomorphological features of BCA, pleomorphic adenoma and adenoid cystic carcinoma.

The radio-imaging of X-ray, CT and MRI for BCA in the parotid gland has been reported in a few studies [12-15]. The present study observed a similar CT scan finding for BCA in the parotid gland as narrated by Chawla et al. [13].

The present case was all the more unique as the skin over the swelling showed hypopigmentation and little scarring as if it’s a swelling in the sub-cutis and not from a lesion within the parotid. It was due to application of some unknown medication over the swelling.

The immunohistochemistry of BCA on the tissue section in the present case showed positive staining for PAN-CK. The diagnostic and confirmative immuno-histochemical marker for BCA is not known. The PAN-CK and CK 5/6 have been quoted to be strongly positive in cells of BCA by Singh et al. [10]. The present study observed strong immuno-reactivity in tumor cells for PAN-CK but immuno-reactivity for S100 was variable as the tumor cells lacked cytoplasmic staining. This finding of immunohistochemistry for BCA of the parotid gland was also reported by Nakabayashi et al. [6] and Singh et al. [10].

The basal cell adenoma that forms a minuscule percentage of neoplasms of the parotid gland is diagnosed rarely on FNAC. Therefore their reports in literature are scarce.

## Conclusions

The basaloid cells with monomorphy with the peculiar pattern of their cell placement prompted the diagnosis of BCA. However, the absence of the heterogenous tissue element and excessive hyaline basement membrane material are a negative diagnostic feature of BCA. The preoperative diagnosis of BCA by FNAC is of significant importance as it decides on the surgical excision and its limits. The cytodiagnosis of BCA in the present case confirms the diagnostic utility of FNAC in salivary gland tumors.
